# Phenotype instability of hepatocyte-like cells produced by direct reprogramming of mesenchymal stromal cells

**DOI:** 10.1186/s13287-020-01665-z

**Published:** 2020-04-10

**Authors:** Iasmim Diniz Orge, Victoria L. Gadd, Judah Leão Barouh, Erik Aranha Rossi, Rejane Hughes Carvalho, Ian Smith, Kyan James Allahdadi, Bruno Diaz Paredes, Daniela Nascimento Silva, Patrícia Kauanna F. Damasceno, Gabriela Louise Sampaio, Stuart J. Forbes, Milena Botelho Pereira Soares, Bruno Solano de Freitas Souza

**Affiliations:** 1 Gonçalo Moniz Institute, FIOCRUZ, Salvador, BA Brazil; 2 Center for Biotechnology and Cell Therapy, São Rafael Hospital, Salvador, BA Brazil; 3 MRC Centre for Regenerative Medicine, Edinburgh, UK; 4 D’Or Institute for Research and Education (IDOR), Rio de Janeiro, RJ Brazil; 5 Institute of Health Sciences, UFBA, Salvador, BA Brazil; 6 National Institute of Science and Technology for Regenerative Medicine, Rio de Janeiro, RJ Brazil; 7 Instituto Gonçalo Moniz, Fundação Oswaldo Cruz, Rua Waldemar Falcão, 121, Candeal, Salvador, Bahia CEP: 40296-710 Brazil

**Keywords:** Hepatocyte-like cells, Direct reprogramming, Mesenchymal stromal cells

## Abstract

**Background:**

Hepatocyte-like cells (iHEPs) generated by transcription factor-mediated direct reprogramming of somatic cells have been studied as potential cell sources for the development of novel therapies targeting liver diseases. The mechanisms involved in direct reprogramming, stability after long-term in vitro expansion, and safety profile of reprogrammed cells in different experimental models, however, still require further investigation.

**Methods:**

iHEPs were generated by forced expression of Foxa2/Hnf4a in mouse mesenchymal stromal cells and characterized their phenotype stability by in vitro and in vivo analyses.

**Results:**

The iHEPs expressed mixed hepatocyte and liver progenitor cell markers, were highly proliferative, and presented metabolic activities in functional assays. A progressive loss of hepatic phenotype, however, was observed after several passages, leading to an increase in alpha-SMA^+^ fibroblast-like cells, which could be distinguished and sorted from iHEPs by differential mitochondrial content. The resulting purified iHEPs proliferated, maintained liver progenitor cell markers, and, upon stimulation with lineage maturation media, increased expression of either biliary or hepatocyte markers. In vivo functionality was assessed in independent pre-clinical mouse models. Minimal engraftment was observed following transplantation in mice with acute acetaminophen-induced liver injury. In contrast, upon transplantation in a transgenic mouse model presenting host hepatocyte senescence, widespread engraftment and uncontrolled proliferation of iHEPs was observed, forming islands of epithelial-like cells, adipocyte-like cells, or cells presenting both morphologies.

**Conclusion:**

The results have significant implications for cell reprogramming, suggesting that iHEPs generated by Foxa2/Hnf4a expression have an unstable phenotype and depend on transgene expression for maintenance of hepatocyte-like characteristics, showing a tendency to return to the mesenchymal phenotype of origin and a compromised safety profile.

## Background

In vitro generation of functional induced hepatocyte-like cells (iHEPs) could overcome some of the hurdles of primary hepatocyte transplantation, including low organ availability and limited proliferative potential provided by current culture protocols. In the past decade, somatic cells, such as fibroblasts or mesenchymal stromal cells (MSCs), have been directly converted into specialized cell types, including hepatocytes [1, 2]. Some of the theoretical advantages of direct reprogramming strategies include the use of less time-consuming protocols, avoidance of the pluripotency stage and generation of cells with increased maturation profile for personalized medicine applications [3]. In fact, iHEPs were generated by direct reprogramming and were shown to be amenable for in vitro expansion and able to repopulate the liver when transplanted into mice [1, 2, 4].

Although different reports indicate the potential safety and efficacy of directly reprogrammed iHEPs to treat certain pre-clinical models of liver diseases, further studies are needed to clarify the mechanisms involved in cell reprogramming and to determine safety and phenotype stability after continuous in vitro expansion, before these cells can be applied in a clinical setting. Transcription factor-mediated direct reprogramming usually combines forced expression of adult hepatocyte genes, such as HNF1 homeobox A (*HNF1A)* or hepatocyte nuclear factor 4 alpha (*HNF4A*) [5], with genes present in early developmental stages, such as forkhead box A2 (*FOXA2*) [6]. Persistent expression of genes involved in early liver development could compromise definitive cell fate, by continuously redirecting the cells towards a progenitor phenotype. Moreover, some protocols of direct reprogramming have generated iHEPs dependent on transgene expression in order to maintain cell identity, reversing to the cell phenotype of origin when exogenous hepatic transcription factor is switched off [7]. Phenotype stability of reprogrammed cells is a crucial issue for clinical applications, considering that billions of in vitro expanded cells would be needed to treat a single patient [8].

Here, we aimed at investigating the safety and phenotype stability of iHEPs after long-term in vitro expansion in culture. The iHEPs were generated by reprogramming mouse bone marrow-derived mesenchymal stromal cells with lentiviral vectors carrying *Foxa2* and *Hnf4a*. The vectors utilized herein allow for a straightforward monitoring of transgene expression through a reporter gene (GFP) and a selectable marker (puromycin resistance) throughout the cell passages. Finally, the safety, efficacy, and fate of iHEPs were evaluated in independent in vivo liver repopulation experiments.

## Materials and methods

### Isolation and culture of primary cells

MSCs were isolated from the bone marrow of male C57Bl/6 mice, as previously described [9]. The cells were cultured in Dulbecco’s modified Eagle’s medium (DMEM) supplemented with 10% fetal bovine serum (FBS) and 1% penicillin/streptomycin (Thermo Fisher Scientific, Waltham, MA, USA), with medium changes every 3–4 days, and maintained in an incubator at 37 °C and humidified atmosphere with 5% CO_2_. After reaching 80–90% confluency, the cells were detached using 0.25% trypsin-EDTA (Sigma-Aldrich, St. Louis, MO, USA) and passaged in a 1:3 ratio.

Fetal hepatoblasts were isolated from mouse fetal liver (E13.5) as described previously [10]. The isolated hepatoblasts were washed and maintained in culture on Matrigel-coated plates with William’s medium E (Thermo Fisher Scientific). Primary hepatocytes were obtained from adult mice by liver enzymatic digestion by a previously described protocol [11].

### Vector production

A 2nd-generation lentiviral system was used, and non-replicative lentiviral particles were produced by transient transfection of HEK293FT cells with psPAX2 (Addgene #12260), pMD2.G (Addgene #12259), and expression vector, at a 2:1:3 ratio, as previously described [9]. Three expression vectors were constructed: pFOXA2IP, pFHIG, and pCWFOXA2. *Foxa2* was amplified from pGCDNsam_Foxa2 (Addgene #33004) with the primers mmFoxa2_BamHI_F and mmFoxa2_BsrGI_R and subcloned between BamHI and BsrGI sites in pEGIP (Addgene #26777). For dox-inducible expression studies, *Foxa2* was amplified with mmFoxa2_NheI_F and mmFoxa2_BamHI_R primers and subcloned into the pCW-cas9 (Addgene # 50661) Tet-on expression vector in the NheI/BamHI flanked region. *Hnf4a* was amplified from pGCDNsam_HNF4α (Addgene #33002) with primers mmHnf4a_XbaI_F and mmHnf4a_BamHI_R and subcloned into pFUWOSKM (Addgene #20328) vector in the XbaI/BamHI flanked region, while IRES-GFP sequence was subcloned in frame, within BamHI/AscI restriction sites, after amplification from MSCVPIG (Addgene #18751) using IRES-GFP_BamHI_F and IRES-GFP_AscI_R primers. Primer sequences are listed in Table S1.

### Generation and expansion of iHEPs

To generate iHEPs, MSCs were transduced with lentiviral vectors expressing *Foxa2* in frame with puromycin resistance gene (pFOXA2IP) and *Hnf4α* in frame with GFP (pFHIG). Transduced cells were cultured in DMEM supplemented with 10% FBS and selected by the addition of 2 μg/mL puromycin to the culture medium 48 h post lentiviral transduction. After 72 h, the medium was replaced with the iHEP culture medium: DMEM/F-12, 10% FBS, 1% penicillin/streptomycin, 0.1 μM dexamethasone (Sigma-Aldrich), 10 mM nicotinamide (Sigma-Aldrich), 1% ITS (Thermo Fisher Scientific), 10 ng/mL FGF-4, 20 ng/mL HGF, 20 ng/mL EGF (Peprotech, Rocky Hill, NJ, USA), and 1 μM SB431542 (Stem Cell Technologies, Vancouver, Canada), on Matrigel-coated dishes (Corning, Corning, NY, USA). To generate iHEPs with inducible *Foxa2* expression vector (pCWFOXA2), 5 μg/mL doxycycline (Sigma-Aldrich) was added to the iHEP medium. The iHEPs were maintained in culture until 90% of confluence was reached and were detached using 2× trypsin solution (Thermo Fisher Scientific). After washing, the cells were resuspended in the iHEP medium and re-seeded using a 1:4 split ratio.

### Liver injury experimental models and iHEP transplantation

All animals received humane care according to the criteria outlined in the “Guide for the Care and Use of Laboratory Animals” prepared by the National Academy of Sciences and published by the NIH. Four- to six-week-old C57Bl/6 male mice were maintained at the animal facility of the Center for Biotechnology and Cell Therapy, São Rafael Hospital, under controlled conditions of temperature (22 ± 2 °C) and humidity (55 ± 10%). The study received prior approval by the local Committee of Ethics for the Use of Animals at São Rafael Hospital, under the protocol number 01/16. Animal experiments performed at the Centre for Regenerative Medicine, Edinburgh, were conducted under procedural guidelines and severity protocols and with ethical permission from the University of Edinburgh Animal Welfare and Ethical Review Body and the UK Home Office. Male and female Ah^cre^Mdm2^fl/fl^ mice were on a C57Bl/6J background, and all animals were housed in specific pathogen-free environment with access to food and water ad libitum.

To model acute-on-chronic disease, male C57Bl/6 mice weighing approximately 20 g were exposed to 10% ethanol diluted in drinking water, for 3 weeks, with access to food ad libitum. At the end of the third week, the animals were fasted for 12 h, with free access to water. Subsequently, 300 μL of a 450 mg/mL acetaminophen (APAP) solution in heated 0.9% saline (40 °C) was administered via i.p. injection. After 4 h, 2 × 10^6^ iHEPs cells were resuspended in 10 μL of Matrigel (Corning) solution (1:25 dilution) and injected intra-hepatically, a protocol that, in a pilot study conducted by our group, resulted in higher engraftment rates when compared to transplantation of iHEPs resuspended in PBS through intrasplenic or intrahepatic routes in the APAP model.

Ah^cre^Mdm2^fl/fl^ mice were used to model chronic liver disease and impaired host hepatocyte cell regeneration [12]. Before transplantation, iHEPs underwent external IMPACT^Tm^ II testing (IDEXX BioAnalytics, Ludwigsburg, Germany) to confirm the absence of infectious agents and *Mycoplasma* contamination. Mice were administered a single dose of β-Naphthoflavone (βNF) at 20 mg/kg via intraperitoneal injection to activate cre recombinase 1 week prior to transplantation. 2 × 10^6^ iHEPs were resuspended in 100 μL PBS and transplanted by intrasplenic injection, while control mice received PBS alone.

### Immunohistochemistry

Tissue was fixed in 10% buffered formalin for 8 h and embedded in paraffin for sectioning. Sections were dewaxed in xylene and rehydrated in decreasing concentrations of ethanol. Tissue underwent heat-induced antigen retrieval for 10 min in Tris-EDTA (pH = 9). For single chromogenic immunodetection, sections were blocked for endogenous peroxidase and avidin/biotin binding sites (Vector Laboratories, Burlingame, CA, USA). Primary antibodies were incubated overnight at 4 °C at the following concentrations: GFP (Abcam, 1:400), HNF4α (Abcam, 1:250), alpha-smooth muscle actin (ɑSMA; Sigma-Aldrich, 1:1000), Foxa2 (Abcam, Cambridge, UK, ab108422, 1:500), albumin (Abcam, 1:100), and epithelial cell adhesion molecule (EpCAM; Abcam, 1:100), and signal was visualized using avidin-biotin complex methods. For immunofluorescent detection, sections were blocked for an hour in protein block (Vector Laboratories) and primary antibodies were incubated overnight and visualized utilizing Alexa Fluor secondary antibodies (Invitrogen, 1:200) and DAPI (1:1000).

### Immunocytochemistry

Cells were fixed with 4% paraformaldehyde (Electron Microscopy Sciences, Hatfield, PA, USA) for 15 min, washed twice with PBS for 5 min, and permeabilized with 0.1% Triton X-100 (Sigma-Aldrich) for 10 min for nuclear antigen labeling. After washing with PBS for 5 min, blocking was performed using background blocker (Dako, Glostrup, Denmark) for 10 min, followed by incubation at 4 °C overnight with the following primary antibodies, diluted in 1% BSA/PBS (Sigma-Aldrich): anti-albumin (1:500, Dako), anti-Foxa2 (1:100, Santa Cruz Biotechnology), anti-CK18 (1:100, Santa Cruz Biotechnology), and anti-e-cadherin (1:100, BD Biosciences, San Jose, CA, USA). On the next day, the following secondary antibodies were used, in 1:500 dilution: anti-mouse IgG Alexa Fluor 488, anti-rabbit IgG Alexa Fluor 568, and anti-goat IgG Alexa Fluor 488 (all from Thermo Fisher Scientific), followed by a 1-h incubation at RT. Nuclei staining was performed with DAPI (Vector Laboratories). Images were captured using an A1+ confocal microscope (Nikon, Tokyo, Japan) or a FluoView 1000 confocal microscope (Olympus, Tokyo, Japan).

### Flow cytometry

For immunophenotyping, MSCs were incubated for 30 min with the following antibodies (diluted 1:100): CD90APC, CD44PE, Sca-1FITC, CD34APC and CD45APC-Cy7 (BD Biosciences), and CD29PE and CD11bPE-Cy5.5 (e-Bioscience, San Diego, CA, USA). At least 50,000 events were collected and analyzed with a Fortessa flow cytometer (BD Biosciences). For fluorescence-activated cell sorting (FACS), 1 × 10^7^ iHEPs were stained with 500 nM MitoTracker Red FM (Thermo Fisher Scientific) following the manufacturer’s instructions, and iHEP MT high and iHEP MT low were sorted using the BD FACS Aria II (BD Biosciences).

### RT-qPCR

Total RNA was extracted using TRIZOL® (Thermo Fisher Scientific) or RNeasy Mini RNA Extraction kit (Qiagen, Hilden, Germany) following the manufacturers’ instructions. RNA integrity was assayed by 1% agarose electrophoresis, and purity was measured photometrically using the NanoDrop™ 1000 (Thermo Fisher Scientific). RNA samples (1 μg) were converted to cDNA using High-Capacity cDNA Reverse Transcription Kit (Thermo Fisher Scientific) or QuantiTect Reverse Transcription (Qiagen). Primer sequences are detailed in Table S1. PCR amplification was performed in an ABI7500 Real-Time PCR System (Thermo Fisher Scientific) under standard thermal cycling conditions. The threshold cycle method of comparative PCR was used to analyze the results. In some experiments, the following commercial primers of Qiagen QuantiTect were used: albumin (*Alb*; Qiagen, QT00115570), alpha-fetoprotein (*Afp*; Qiagen, QT00174020), *Epcam* (Qiagen, QT02304456), Sca-1 (*Ly6a*; Qiagen, QT00293167), cytokeratin 19 (*Krt19*; Qiagen, QT00156667), *Hnf4a* (Qiagen, QT00144739), peptidylprolyl isomerase A (*Ppia*; Qiagen, QT00247709), and in RT-qPCR was performed in a LightCycler 480 II equipment (Roche). Gene expression was normalized using the endogenous PPIA gene, and the samples were amplified in triplicate. The threshold cycle method of comparative PCR was used to analyze the results.

### Transmission electron microscopy

The iHEPs and MSCs were grown in 24-well plates adhered in Matrigel-coated plastic coverslips (Corning) suitable for ultra-thin cuts until reaching 90% confluency. After this, the cells were fixed for 1 h at RT with 1% osmium tetroxide/0.8% potassium ferrocyanide solution (Sigma-Aldrich). Subsequently, the material was dehydrated in increasing concentrations of acetone (30, 50, 70, 90, and 100%) and included in polybed resin (Polysciences, Washington, PA, USA). Ultra-thin sections were obtained from a UC732 ultramicrotome (Leica Microsystems, Wetzlar, Hesse, Germany) and collected on 300-mesh copper grids, contrasted with uranyl acetate and lead citrate, and observed on a transmission electron microscope (JEOL JEM-1230) at 15 kV.

### Functional analyses

For PAS staining, the cells were fixed in a 1:1 acetone/methanol solution at − 20 °C for 20 min and washed twice with distilled water. Then, the cells were incubated in 1% periodic acid solution for 10 min at RT. After two washes with distilled water, Schiff reagent was added and incubated for 30 min at RT. Finally, distilled water heated at 40 °C was used to wash the reagent.

For the indocyanine green (ICG) uptake assay, an ICG solution (Sigma-Aldrich) was added to the cultured cells at a final concentration of 1 mg/mL. The cells were incubated at 37 °C for 1 h and washed three times with PBS, and then cellular uptake of ICG was examined. Next, to induce the cellular release of ICG, cells were incubated in culture medium without ICG solution at 37 °C for 4 h. For low-density lipoprotein (LDL) uptake assay, the cells were incubated with 10 μg/mL acetylated LDL labeled with 1,1′-dioctadecyl-3,3,3′,3′-tetramethylindo-carbocyanine perchlorate, DiI (DiI-Ac-LDL) (Thermo Fisher Scientific) for 4 h at 37 °C, followed by DAPI staining. For visualization of lipid inclusions, the cells were fixed in 4% PFA for 15 min at RT, washed in distilled water, and incubated in 70% ethanol for 3 min. Finally, cells were stained with Oil red O solution (Sigma-Aldrich) for 5 min and washed with 70% ethanol.

For assessing the expression levels of Cyp450 enzymes, the cells were incubated with dexamethasone (100 μM), rifampicin (30 μM), omeprazole (50 μM), or phenobarbital (1 mM) for 48 h at 37 °C. Then, the expression of the genes encoding the cytochrome P450, family 3, subfamily a, polypeptide 11 (*Cyp3a11*); cytochrome P450, family 3, subfamily a, polypeptide 44 (*Cyp3a44*); and cytochrome P450, family 1, subfamily a, polypeptide 1 (*Cyp1a1*) enzymes was evaluated by RT-qPCR (Table S1). For the evaluation of CYP3A4 enzyme activity, the P450-Glo CYP3A4 assay system kit (Promega, Madison, WI, USA) was used according to the manufacturer’s protocol. After incubation at RT protected from light for 30 min, luminescence was measured on the GLOMAX 20/20 Luminometer reader (Promega). The luminescence data were normalized by the respective total protein concentration per well.

The bipotent nature of iHEPs was assessed by evaluation of morphology and gene expression after incubation with either iHEP media supplemented with the small molecules 0.5 mM valproic acid, 5 μM parnate, and 1 μM TTNPB, or with the maturation media (MM): DMEM F12, 10% SBF, 1% penicillin/streptomycin, 10 μM dexamethasone, 10 μM SB431542, 20 ng/ml oncostatin M, 0.5 mM valproic acid, 5 μM parnate, and 1 μM TTNPB. Qiagen QuantiTect commercial primers were used: *Alb* (Qiagen, QT00115570), *Afp* (Qiagen, QT00174020), aquaporin 1 (*Aqp1*) (Qiagen, QT00109242), and *Ppia* (Qiagen, QT00247709). Gene expression was normalized using the endogenous gene *Ppia*, and samples were amplified in triplicate.

### Statistical analysis

Data were analyzed using Student’s *t* test or one-way ANOVA followed by Tukey or Newman-Keuls multiple comparison tests. Log-rank (Mantel-Cox) test was performed for comparison of survival curves. All data were analyzed using GraphPad Prism v.5.0 software (GraphPad Inc., San Diego, CA, USA). Differences were considered statistically significant for *p* values < 0.05.

## Results

### Sustained, high Foxa2 expression is necessary for direct conversion of MSCs to iHEPs

**Fig. 1:**
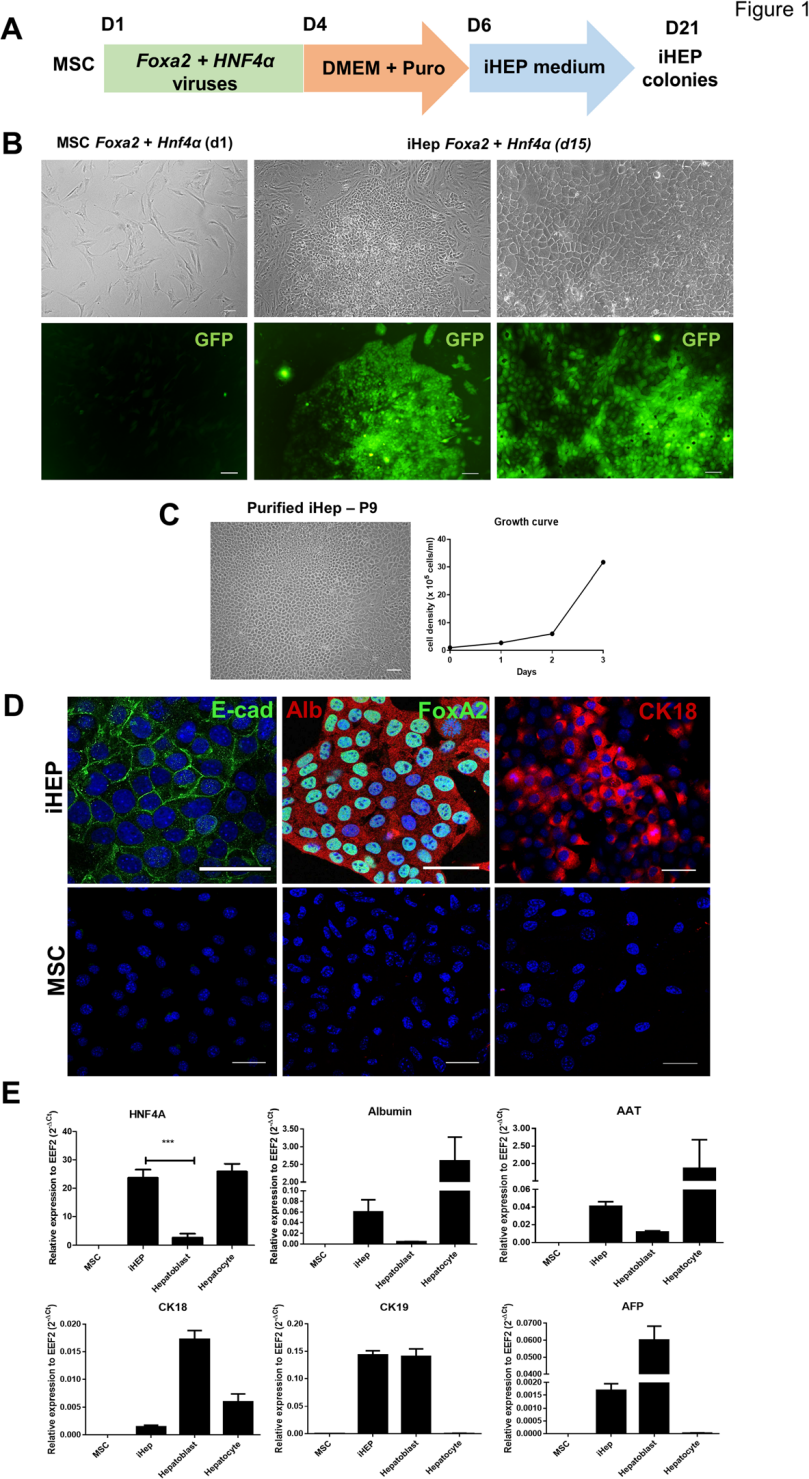
Generation and phenotypic characterization of iHEPs. Schematic diagram depicting the procedure and time-course for the direct conversion of MSCs into iHEPs (**a**). Phase-contrast and fluorescence microscopy images representing MSCs’ morphology before hepatic reprogramming and, at reprogramming day 15, the appearance of colonies of epithelial-like cells with GFP expression (**b**). Phase-contrast image of a purified iHEP culture and respective growth curve at P8 (**c**). Characterization of iHEPs by immunofluorescence showing expression of E-cadherin and FoxA2 (green) and albumin and CK18 (red). Nuclei are visualized in blue by DAPI staining. Wild-type MSCs were used as controls (**d**). RT-qPCR analysis of MSCs, iHEPs, fetal hepatoblasts (E13.5), and adult primary hepatocytes for hepatic markers (**e**). Data are shown as mean ± SEM of three independent samples for each group. *p < 0.05; **p < 0.01; ***p < 0.001. Scale bar 100 μm

The MSCs’ identity was confirmed by plastic adherence, fibroblastic morphology, and potential to differentiate into adipocytes, chondrocytes, and osteocytes (Fig. S1A). The cells were positive for the MSCs’ markers CD90, CD44, CD73, CD29, and Sca-1 and negative for hematopoietic markers CD19, CD34, and CD117 (Fig. S1B). Next, we conducted the reprogramming protocol (Fig. 1a) and transduced MSCs with lentiviral vectors for constitutive expression of *Foxa2* and *Hnf4a*. Treatment with puromycin for 72 h allowed for the selection of the cells that were successfully transduced with *Foxa2* lentivirus, while GFP expression was used as a reporter to detect the cells also transduced with *Hnf4a* lentivirus (Fig. 1b).

After 15 days in culture with iHEP medium, large colonies of GFP^+^ epithelial-like cells (iHEPs) were observed and expanded in vitro (Fig. 1b). The cells presented a high proliferative rate and were purified from the cells with fibroblastic morphology by successive passages (Fig. 1c, d). Similar results were obtained by reprogramming MSCs from three different isolates. Once visually homogeneous cultures of epithelial-like cells were obtained, the cells were characterized. Foxa2 expression was confirmed by immunostaining and positive expression of the epithelial marker e-cadherin, and hepatocyte markers albumin and CK-18 were also observed (Fig. 1e). RT-qPCR analysis revealed a higher expression of hepatic markers albumin and AAT in iHEPs compared to fetal hepatoblasts (E13.5) and parental MSCs, but at lower levels than primary hepatocytes. The iHEPs also expressed the early hepatic markers *Afp* and *Ck18* at lower levels than fetal hepatoblasts. While *Hnf4a* expression levels in iHEPs were similar to adult hepatocytes, an increased expression of the immature bipotent progenitor marker *Krt19* was found in iHEPs (Fig. 1f–k).

**Fig. 2:**
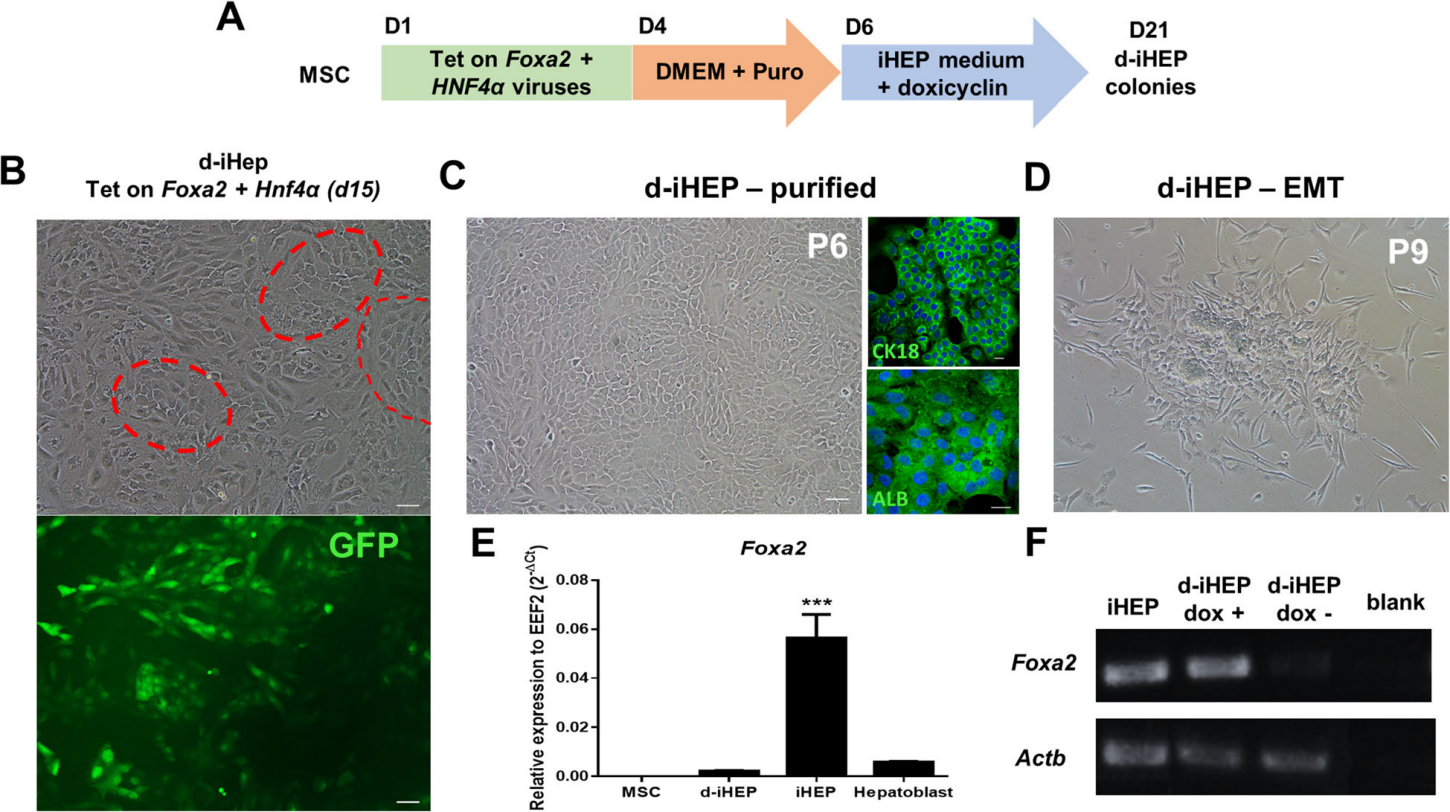
Direct reprogramming of MSCs to d-iHEPs using dox-inducible *Foxa2*. Schematic diagram depicting the procedure and time-course for the direct conversion of MSCs into d-iHEPs (**a**). Colonies of epithelial-like d-iHEPs (red dashed circles) with GFP expression are observed after 15 days of iHEP medium exposure (**b**). Purified d-iHEPs at passage 6 expressing the hepatic markers albumin (ALB) and CK18 (green) by immunofluorescence. Nuclei are visualized in blue by DAPI staining (**c**). The increased presence of fibroblast-like cells after continuous passaging in the absence of doxycycline (**d**). RT-qPCR showing decreased levels of *Foxa2* mRNA in d-iHEPs when compared to iHEPs (**e**). Validation of Tet-on *Foxa2* system by analysis of Foxa2 expression in iHEPs and d-iHEPs in the presence or absence of doxycycline, by RT-PCR (**f**). ***p < 0.001. Scale bars 50 μm

Since *Foxa2* expression is high during liver bud formation but falls between E12.5 and E15.5 before increasing again in the adult liver [13], we hypothesized that persistently high *Foxa2* transgene expression could influence and possibly impair hepatocyte maturation in iHEPs. In order to evaluate whether iHEPs could be generated by transient expression of *Foxa2*, MSCs were transduced with a dox-inducible *Foxa2* expression vector, along with the *Hnf4a* constitutive expression vector. The addition of doxycycline to the MSC’s culture medium after puromycin selection successfully induced the expression of *Foxa2* (Fig. 2a). Smaller and less frequent epithelial-like colonies were obtained, compared to the previous protocol (Fig. 2b). These cells (d-iHEPs) were purified within 6 passages and were found to express CK18 and albumin, as shown by immunofluorescence (Fig. 2c), and *Foxa2* at similar levels to fetal hepatoblasts, but at lower levels when compared to iHEPs, as shown by RT-qPCR (Fig. 2e). Moreover, the cells presented a heterogeneous morphology and shifted back to a fibroblastic morphology after continuous passaging, cryopreservation, and thawing, or after doxycycline removal (Fig. 2d, f). Since expandable d-iHEPs were not obtained, the next steps were performed using the iHEPs generated with *Foxa2* constitutive expression system.

### iHEPs exhibit hepatocyte functions

**Fig. 3:**
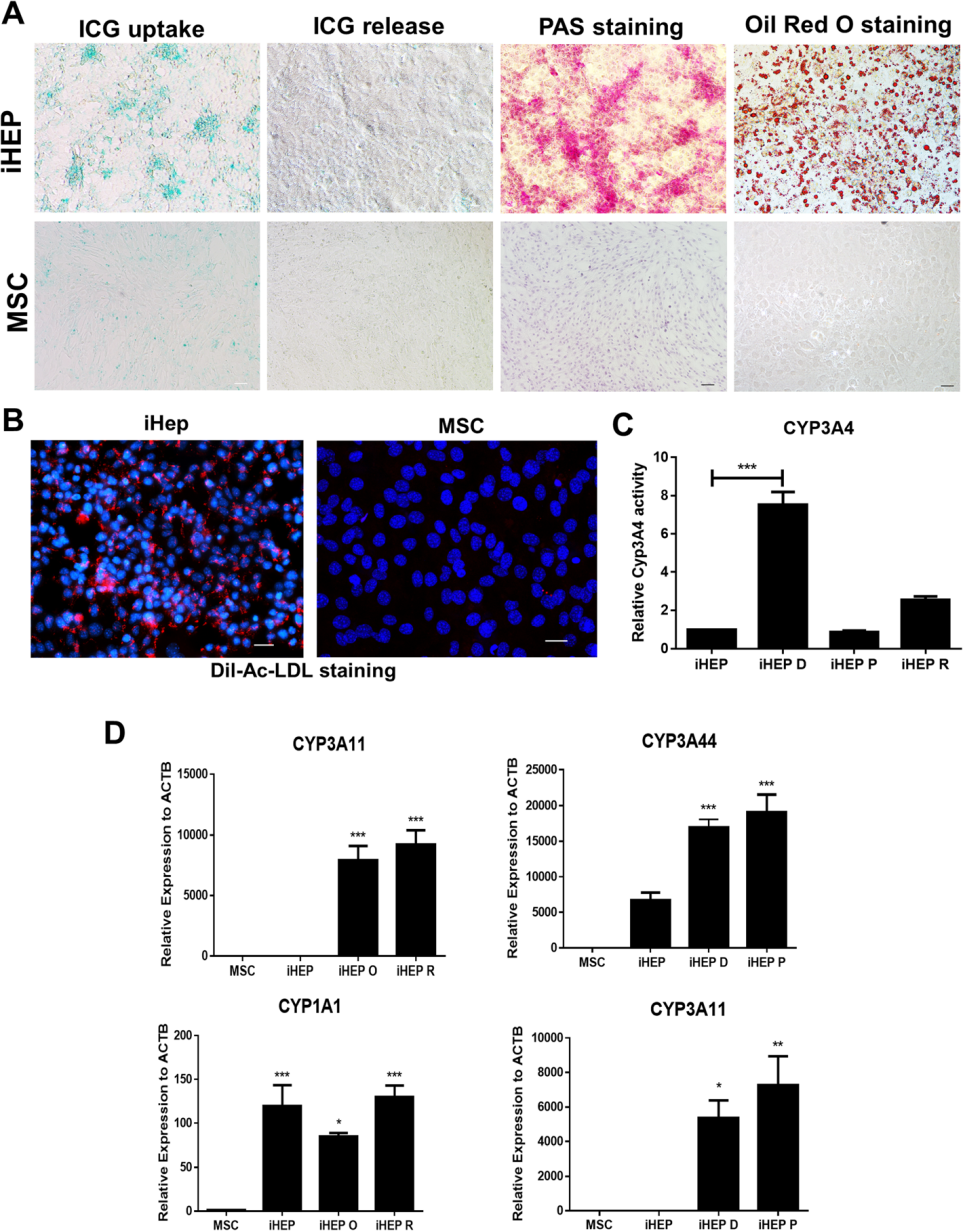
iHEPs display hepatocyte functions. Functional evaluation of iHEPs compared to MSCs, by ICG uptake and release, PAS staining, and Oil red O staining (**a**). LDL uptake evaluation (Ac-LDL, seen in red fluorescence), nuclei stained with DAPI (blue) (**b**). Evaluation of Cyp3a4 activity in response to 100 μM dexamethasone (iHEP D), 30 μM rifampicin (iHEP R), or 1 mM phenobarbital (iHEP P) (**c**). RT-qPCR analysis of mRNA expression levels of Cyp450 enzymes in response to 100 μM dexamethasone, 30 μM rifampicin, 1 mM phenobarbital, or 50 μM omeprazole (iHEP O). Expression levels were compared with non-induced iHEPs and MSCs (**d**)

After the detection of hepatic markers, the functional activities of iHEPs were evaluated. ICG clearance can be used for functional analysis of the hepatocyte, since primary hepatocytes are able to capture ICG and excrete it in the bile [14]. The iHEPs were able to uptake ICG after 1-h incubation and release it 4 h after. Glycogen and lipid storage capacities were detected in iHEPs by PAS and Oil red O staining, respectively (Fig. 3a). Cholesterol uptake was also demonstrated by using a low-density acetylated lipoprotein (Ac-LDL) labeled with a fluorescent probe (Fig. 3b). Drug metabolism ability of iHEPs was evaluated by gene expression analysis, showing upregulation of *Cyp3a11*, *Cyp1a1*, and *Cyp3a44* in iHEPs after incubation with dexamethasone, phenobarbital, rifampicin, or omeprazole. We also detected increased CYP3A4 activity after exposure of iHEPs to the drugs (Fig. 3c, d).

### Epithelial-to-mesenchymal transition of iHEPs in vitro

**Fig. 4:**
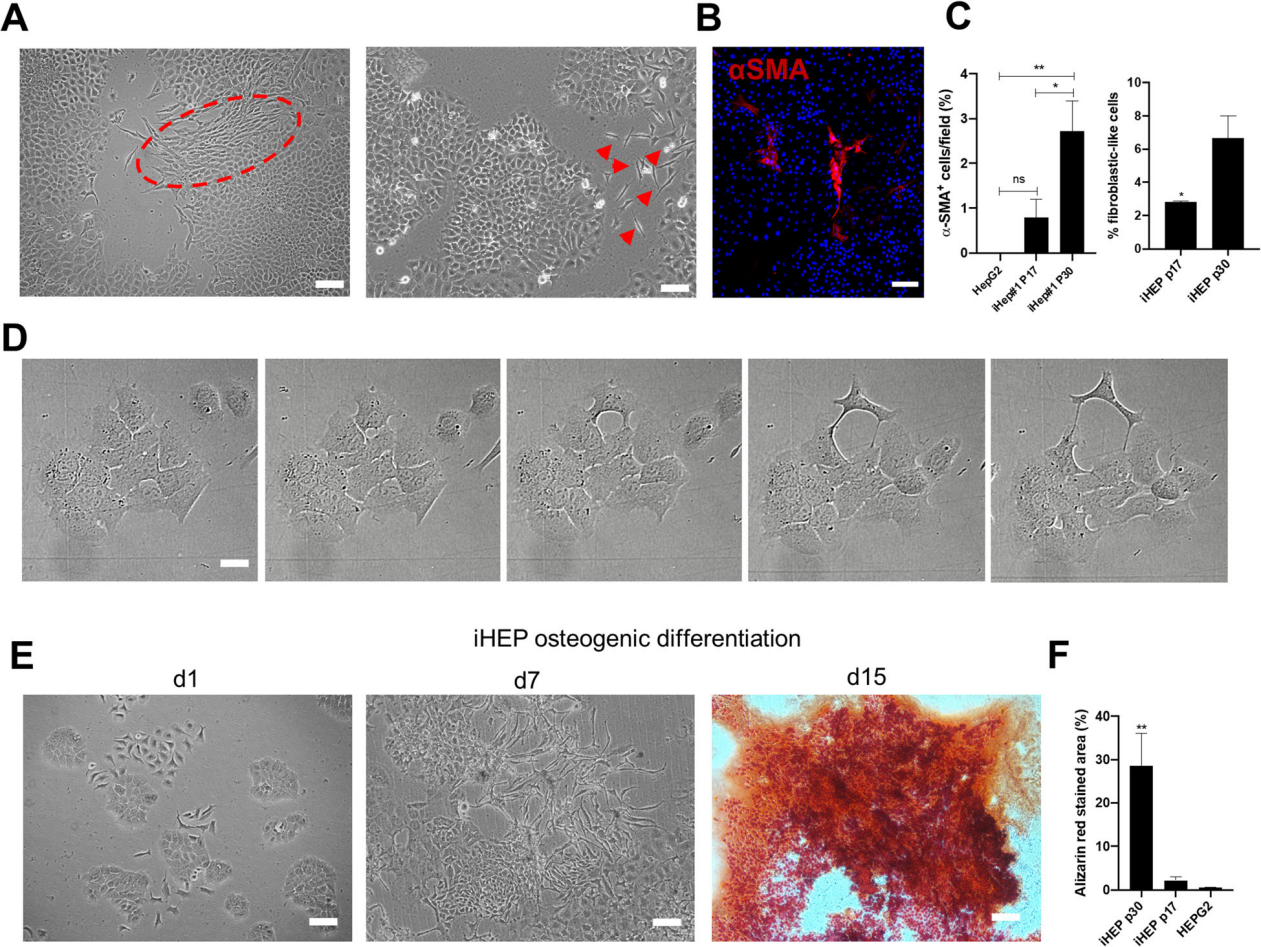
Long-term expansion of iHEPs is associated with phenotype loss by EMT. Phase-contrast microscopy images obtained from iHEP cultures at p30, showing the presence of spindle-shaped cells (red dashed circle and arrowheads) surrounding epithelial cells (**a**). Confocal microscopy image showing ɑSMA staining (red) in spindle-shaped cells (**b**). Quantification of fibroblast-like cells based on ɑSMA staining and high-content image morphology analysis, comparing early- and late-passage iHEPs, using HepG2 hepatoma line as control (**c**). Sequential time-lapse stills showing a fibroblast-like cell emerging from iHEP colony (**d**). Images of late iHEPs stimulated with osteogenic differentiation medium evaluated on D1 and D7 (phase-contrast microscopy) and stained for calcium-rich matrix with Azilarin red, at D15 (**e**). Quantification of Alizarin red staining in early and late iHEPs, using HepG2 hepatoma cell line as control. Bars 100 μm. *p < 0.05; **p < 0.01

In order to evaluate phenotype stability and determine the long-term fate of iHEPs in vitro, the cells were maintained in culture for successive passages and cell morphology was periodically evaluated. Although CK18, albumin, and e-cadherin were detected both in early- and late-passage iHEPs, an increased presence of spindle-shaped cells positive for the myofibroblast marker a-SMA was found surrounding epithelial-cell like colonies (Fig. 4a–c). We hypothesized that these cells could be remnants of non-reprogrammed MSCs, expanded as culture contaminants due to ineffective iHEP purification, or alternatively, that these cells could be derived from iHEPs that reversed to a mesenchymal phenotype in a process of epithelial-mesenchymal transition (EMT). Time-lapse analysis demonstrated that iHEPs indeed lose their epithelial morphology, detaching from neighboring cells in the colony and acquiring a fibroblast-like morphology (Fig. 4d). Moreover, low- or high-passage iHEPs were incubated in osteogenic differentiation media and substantial cell death was observed, while surviving cells changed morphology and formed areas that were positively stained with Alizarin red, confirming osteogenic potential (Fig. 4e, f). Moreover, alizarin red-stained area was significantly higher in high-passage iHEPs, when compared to low-passage iHEPs (Fig. 4f).

### Mitochondrial content reflects different cell populations in late iHEP cultures

Ultrastructural analysis showed that iHEPs have an increased mitochondrial content compared to MSCs (Fig. S2A). Therefore, we hypothesized that differential mitochondrial content could be utilized to sort iHEPs from contaminating MSCs by using the viable cell-compatible fluorescent probe targeting mitochondria (Mitotracker). We found that iHEPs present an intermediate level of Mitotracker staining compared to MSCs, which showed low-level intensity, and to primary hepatocytes, which presented a high-level intensity (Fig. S2B). We also observed a population with low fluorescence level within iHEPs, which may correspond to MSC remnants or cells that underwent EMT.

**Fig. 5:**
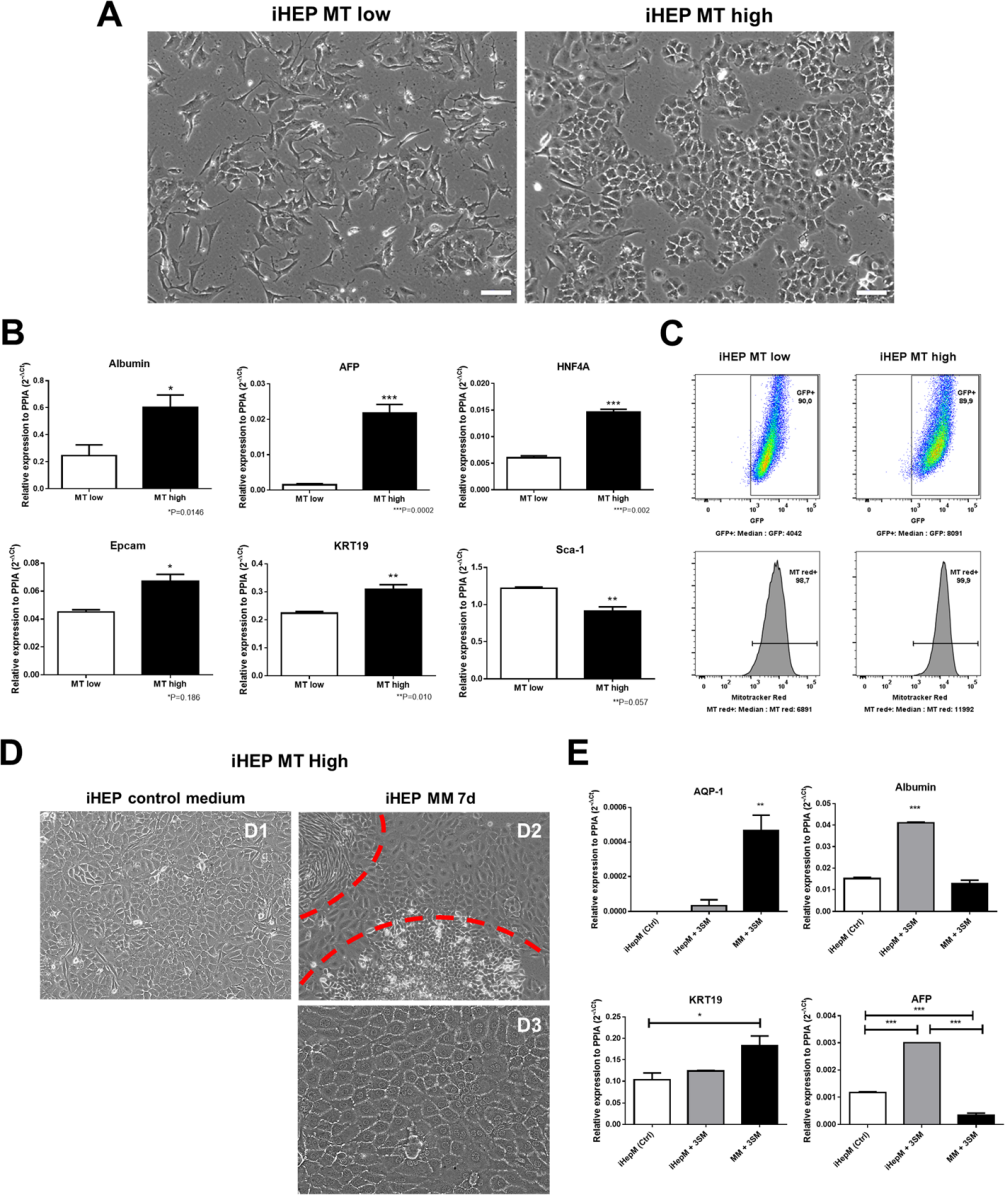
Mitochondrial content reflects different subpopulations in late iHEP cultures. Phase-contrast images obtained from iHEP cultures after mitochondrial content-based cell sorting for isolation of iHEP MT^High^ and iHEP MT^Low^ supopulations. Bars 100 μm (**a**). RT-qPCR analysis of albumin, AFP, HNF4α, Epcam, KRT19, and Sca-1 mRNA levels, comparing the two populations (**b**). Relation between intensity of GFP fluorescence (*Hnf4*α-reporter) and MitoTracker staining. MT^High^ and MT^Low^ iHEPs were labeled with MitoTracker Red FM and analyzed by flow cytometry (**c**). Morphological alterations observed by phase-contrast microscopy in iHEP MT High subpopulation after being exposed to maturation media for 7 days. Bar 100 μm (**d**). RT-qPCR analysis for quantification of aquaporin-1, Afp, and albumin mRNAs after maturation protocols. *p < 0.05; **p < 0.01; ***p < 0.001 (**e**)

To further investigate the characteristics of these different subpopulations, the cells were sorted based on Mitotracker staining into populations with high and low intensities (iHEP MT^High^ and iHEP MT^Low^). We found significant morphological differences between these populations, with iHEP MT^High^ cells presenting epithelial-like morphology and iHEP MT^Low^ presenting a fibroblast-like morphology (Fig. 5a). GFP expression was also higher in the iHEP MT^High^ population, along with increased gene expression of *Hnfa*, *Alb*, *Afp*, *Epcam*, and *Krt19* (Fig. 5b) and reduced expression of *Sca1* and GFP (Fig. 5b, c).

In order to confirm the bipotent nature of iHEPs and to determine whether these cells could be induced to mature in vitro, we incubated iHEP MT^High^ cells with the addition of valproic acid, parnate, and TTNPB small molecules in the culture media or with a maturation media (MM) also supplemented with the same small molecules. After maintaining the cells for 7 days culture on MM, culture heterogeneity increased when compared to control (Fig. 5 (D1)), with the presence of cells with different morphologies, predominantly composed of small cells with flattened or cuboidal morphology (Fig. 5 (D2)), but also including islets of cells that resemble hepatocytes (Fig. 5 (D3)), presenting high cytoplasm/nucleus ratio, epithelial cell junctions, dense cytoplasm, and binucleation (Fig. 5d). Gene expression analysis demonstrated that incubation with the maturation media was associated with upregulation of either cholangiocyte or hepatocyte markers (Fig. 5e).

### iHEP fate is highly influenced by in vivo signaling

**Fig. 6:**
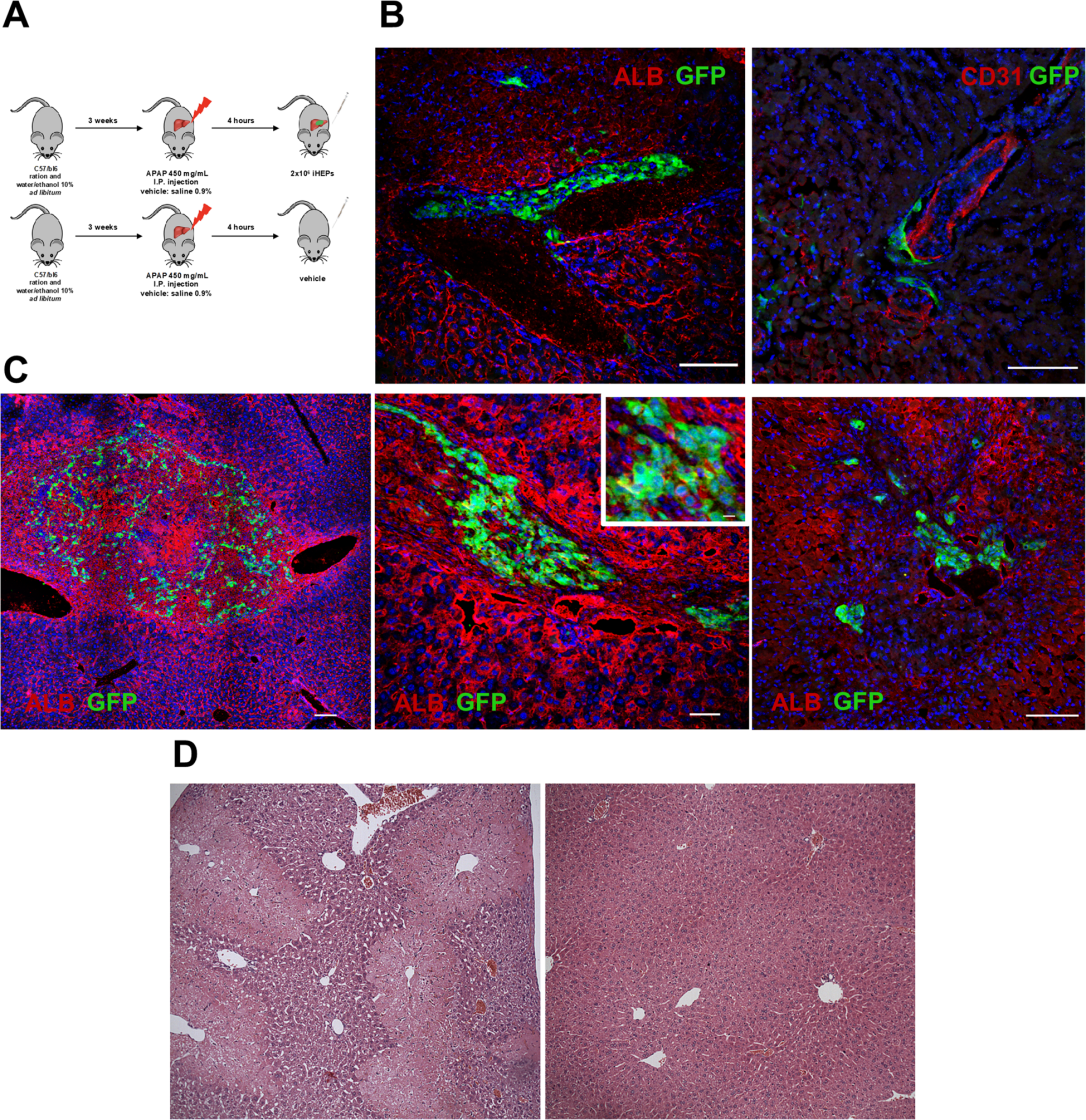
Evaluation of liver repopulation capacity of iHEPs in the APAP acute liver injury model. Experimental design (**a**). Confocal microscopy of liver sections obtained from iHEP-transplanted mice 48 h (**b**) or 7 days (**c**) after cell transplantation. iHEPs are visualized by GFP expression (green), while albumin or CD31+ vessels are seen in red. Nuclei were stained with DAPI (blue). Inset shows a detail for observation of Alb/GFP double staining. Scale bars 100 μm (**b**, **c**; right), 200 μm (**c**; left), 50 μm (**c**; middle). Representative images of H&E-stained liver sections showing extensive areas of centrolobular necrosis 48 h after APAP injection (**d**; left) and complete restoration of normal architecture 7 days after APAP injection (**d**; right), with no signs of ectopic proliferation

To evaluate the in vivo cell fate and liver repopulation ability of iHEPs, we induced an acute-on-chronic liver injury mouse model caused by APAP intoxication following ethanol pretreatment (Fig. 6a). Mice were euthanized either 2 or 14 days after cell infusion for cell tracking. The iHEPs were found in 3 out of 5 mice, in the intravascular space, lining endothelial cells, 2 days after infusion (Fig. 6b). After 14 days, however, most cells had crossed the endothelial barrier and entered the liver parenchyma, but with limited repopulation (Fig. 6c). Of the engraftment cells, albumin expression was present, but appeared to be lower than resident hepatocytes (Fig. 6c). Widespread centrilobular necrosis was seen 2 days after APAP injection; however, after 2 weeks, the liver had completely regenerated, and no morphological difference was observed between iHEP-treated and PBS-treated mice (Fig. 6d). No signs of tumors or ectopic tissue formation were observed.

**Fig. 7:**
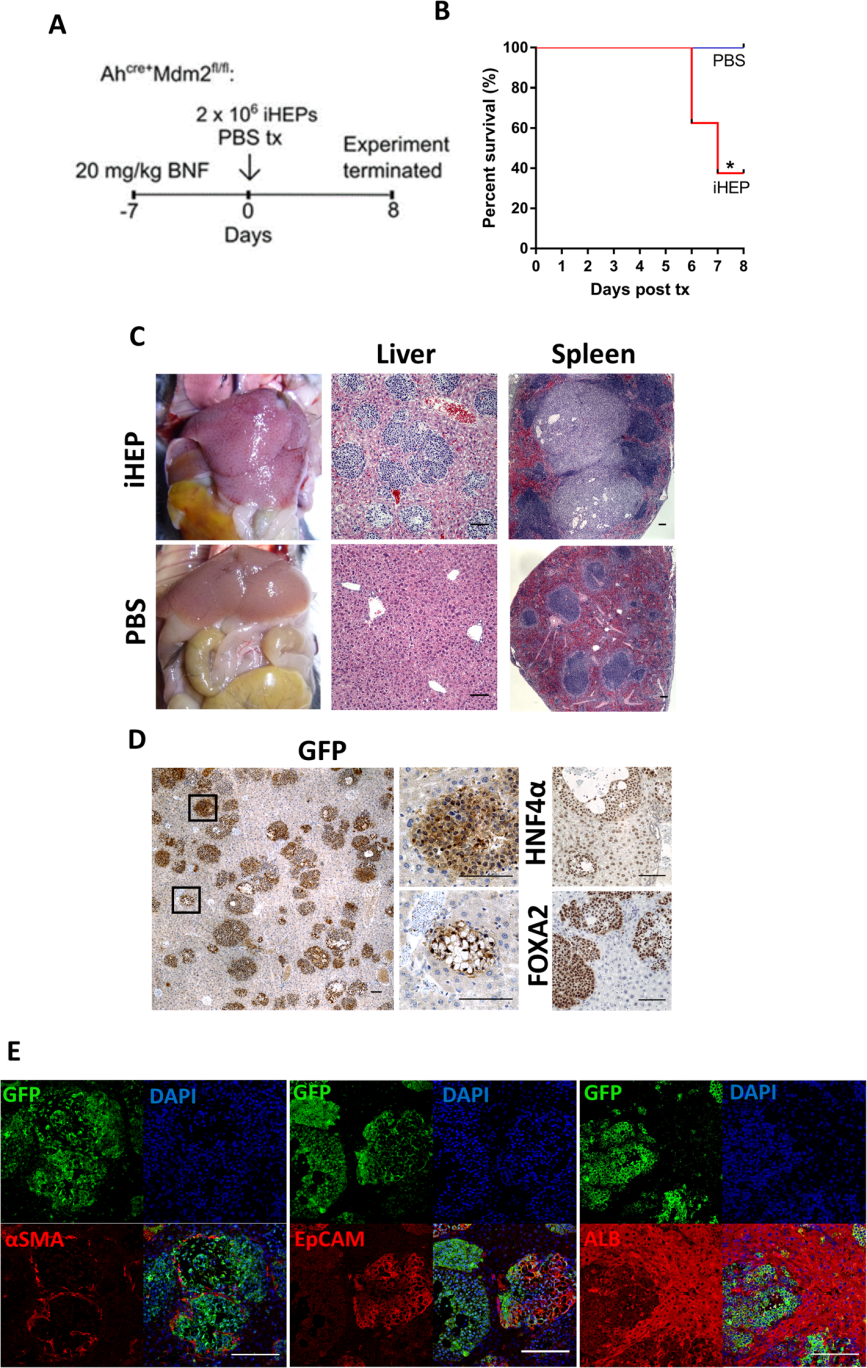
Experimental schematic for iHEP transplantation into the Ah^Cre^Mdm2^fl/fl^ mouse model (**a**). Kaplan-Meier survival plot (**b**). Gross morphology and comparative H&E staining of the liver and spleen of iHEP vs PBS transplanted mice (**c**). Confirmation of iHEP engraftment showing expression of GFP (small boxes represent magnified regions), Foxa2, and HNF4ɑ (**d**). Characterization of engrafted iHEPs utilizing dual immunofluorescent staining for GFP and ɑSMA, EpCAM, or albumin (**e**). *p < 0.05. Scale bars 200 μm

Mouse donor hepatocytes are known to rapidly re-enter cell cycle, proliferate, and are the major contributors to hepatic regeneration during acute liver injury [4]. Therefore, we performed additional in vivo experiments using the Ah^Cre^Mdm2^fl/fl^ transgenic mouse model, which, following induction of hepatocyte senescence, apoptosis, and necrosis through conditional MDM2 deletion, favors liver repopulation by transplanted cells [12]. Seven days post-transplantation, > 60% of mice that received iHEPs were found dead despite showing no prior clinical signs, and by day 8, the experiment was terminated for humane reasons. None of the control PBS-treated mice had become ill for the duration of the experiment but were also euthanized for comparison (Fig. 7a, b). Such sudden death is suggestive of fulminant liver failure associated with venous occlusion and resulting congestive hepatopathy, as observed at necropsy of iHEP-treated mice (Fig. 7c). In striking contrast to the acute liver injury model, GFP^+^ iHEPs were identified in all transplanted mice with 6 out of 8 mice demonstrating extensive engraftment. Large islands of cells were identified by H&E staining in the liver and spleen (Fig. 7c), and immunostaining with GFP confirmed the iHEP origin (Fig. 7d). The iHEP colonies were heterogeneous, displaying epithelial-like morphologies, adipocyte-like morphologies, or a combination of both (Fig. 7d). Further characterization was performed to assess how in vivo signaling may have affected iHEP phenotype. The iHEP colonies retained forced overexpression of HNF4ɑ and Foxa2, were largely positive for K19, and contained a number of ɑSMA-positive cells (Fig. 7d). Interestingly, co-staining with EpCAM highlighted a population of dual-positive cells, suggesting that a small proportion showing progenitor-like characteristics (Fig. 7d). Similar to APAP-injured mice, iHEPs were found to express albumin at lower levels than resident hepatocytes (Fig. 7e).

## Discussion

In the present study, we successfully generated expandable iHEPs by direct reprogramming MSCs by forced expression of the transcription factors *Foxa2* and *Hnf4a*. Although the iHEPs produced expressed hepatocyte genes and displayed functional activity in vitro, these cells were shown by long-term culture to present an unstable identity and a tendency to return to a mesenchymal phenotype. Importantly, these cells failed to mature in vivo in a transgenic mouse model designed for liver repopulation studies, showing extensive proliferative activity and reversed into cells of mesenchymal origin, with detrimental effects to the mice.

Lineage conversion by forced transcription factor expression has challenged the concepts of cell plasticity since its pioneer description, in 1987 [15]. In the past years, different combinations of transcription factors, soluble factors, and small molecules were shown to be effective in generating functional iHEPs [1, 2, 4, 16–25]. Here, we employed the combination of *Foxa2* and *Hnf4a*, which reproducibly and effectively led to the generation of iHEP colonies. Interestingly, the same factors were shown, by in silico analysis of six gene expression databases from independent studies, to regulate the expression of a significant number of common differentially expressed genes during hepatic direct reprogramming in all samples, in both mouse and human cells, irrespectively of the combination of exogenous transcription factors utilized [26].

Although the iHEPs generated herein displayed some degree of hepatocyte function, the gene expression profile differed from primary hepatocytes, showing a mixed expression of immature progenitors and mature hepatocyte markers. This is in accordance with previous observations, which also report differences at the epigenetic level comparing iHEPs and hepatocytes [4, 17, 19]. While extensive work has been done to understand the molecular mechanisms involved in somatic cell reprogramming to pluripotency, including the erasure and remodeling dynamics of epigenetic marks, little is known about direct reprogramming [4, 27, 28]. Future studies are necessary to explore the changes of the global epigenome if directly reprogrammed cells are to be used in clinical studies. Furthermore, expanded analyses, beyond a panel of phenotypic makers, gene expression, and standard functional tests may be required to adequately classify reprogrammed cells and improve lineage conversion protocols. For instance, deeper analysis of iHEPs reprogrammed from fibroblasts revealed an unexpected hindgut identity—or endodermal progenitor—in these cells [28].

Here, we demonstrated that sustained expression of both *Foxa2* and *Hnf4a* is necessary to reprogram and maintain the iHEPs’ phenotype, since high transgenic *Hnf4a* expression, tracked by GFP reporter, was associated with increased hepatocyte gene expression and time-controlled reduction of transgenic *Foxa2* expression led to a reversion to a mesenchymal phenotype. These results suggest that MSCs were not fully reprogrammed and hepatocyte-like characteristics acquired by these cells are dependent on sustained expression of the exogenous transcription factors. Similar results were recently described for human iHEPs generated using a *HNF1A*-based protocol [7].

MSCs are a good cell source for application in cell therapy, as they are easy to obtain and expandable in culture and continue to gain safety data from clinical trials [29]. We hypothesized that MSCs could be reprogrammed to iHEPs with high efficiency, which was confirmed by our data, in accordance with previous reports [2, 30]. Here, the iHEP colonies were all composed by cells that were successfully transduced with both *Foxa2* and *Hnf4α*, and no colonies appeared in MSCs transduced with a single transcription factor or only stimulated with the iHEP medium.

As well as ensuring identity, purity is one of the minimal criteria for cell-based products [31]. In comparison to iPSCs, which can be clonally expanded indefinitely, iHEPs present a high proliferative activity and are usually expanded from a bulk population [32]. Here, we report a method for purification of reprogrammed cells based on mitochondrial staining, advantage of the known higher amounts of mitochondria present in hepatocytes, when compared with stromal cells [33]. Interestingly, in vitro dedifferentiation of primary hepatocytes—a process that has been well known for many years—is associated with large-scale downregulation of mitochondrial proteins [34].

Safety and liver repopulation ability of iHEPs has been demonstrated in different studies, using the Fah^−/−^ mouse model [1, 2, 4, 16, 30]. Relatively limited efficacy, however, was found when compared to primary hepatocytes. The main advantage of using Fah^−/−^ model is the induction of a selective pressure that favors liver repopulation by transplanted cells. However, this model is used to mimic type I tyrosinemia, a very rare human disease, and does not recapitulate the complex environment of many human liver diseases. Therefore, transplantation of iHEPs should be performed in animal models that represent a spectrum of human liver disease to ensure our understanding of how these cells respond to various environmental cues. To investigate liver repopulation ability and safety of iHEP transplantation, we used here the APAP-induced acute liver injury model, as well as the Ah^Cre^Mdm2^fl/fl^ mouse model, which recreates the widespread hepatocyte senescence commonly seen in human chronic liver diseases [12]. Surprisingly, and in contrast to the findings of the APAP acute liver injury model, uncontrolled and extensive iHEP proliferation was observed, exceeding severity limits within a few days. Previously, the same model was used to prove the regenerative capacity of transplanted hepatic progenitor cells to restore the liver parenchyma, with no adverse events [12]. These results highlight the significant influence of tissue microenvironment on engraftment, proliferation, and consequent phenotype of candidate cell therapies. The regenerative niche of induced Ah^Cre^Mdm2^fl/fl^ mice, which previously assisted in the differentiation and maturation of primary hepatic progenitor cells into hepatocytes, caused iHEPs to hyper proliferate and generate an undefined phenotype. Understanding the unique tissue microenvironments of various disease etiologies may aid in identifying the mechanisms that dictate somatic cell reprogramming and maturity, with the potential to generate functional cells for clinical cell therapy and avoid phenotypic instability. Importantly, it is essential that both the therapeutic capacity of candidate cell types and the condition of recipient liver is considered to ensure safe and effective cell therapy.

## Conclusion

In summary, the present study revealed that *Foxa2*/*Hnf4a*-mediated direct reprogramming of MSCs led to the production of expandable iHEPs, which express markers of both mature and immature hepatocytes and some degree of hepatic function. In vitro expansion of iHEPs, however, showed that these cells are not fully reprogrammed, depend on high expression of exogenous transcription factors, and present a plastic identity, with a tendency to return to a mesenchymal phenotype. Moreover, in vivo application of such cells raised safety concerns, due to uncontrolled cell proliferation, widespread liver engraftment, and generation of both hepatic and ectopic mesenchymal derivatives. These results suggest that further direct reprogramming protocol optimizations are needed for proper generation of cells that resemble hepatocytes, along with careful evaluation and deeper characterization of iHEPs, with special attention to the safety evaluation in different animal models, before considering any translational cell therapy applications.

## Supplementary information

**Additional file 1 : Figure S1**. Characterization of mesenchymal stromal cells. MSCs’ morphology seen by phase-contrast microscopy (A), and representative images of tri-lineage differentiation assays (B) showing positive staining for Oil Red (adipogenic), Alizarin Red (osteogenic) and Alcian Blue (chondrogenic). Flow cytometry analysis with a panel of MSCs and hematopoietic cell markers (C).

**Additional file 2 : Figure S2.** Differences in mitochondrial content among iHEPs, MSCs and hepatocytes. Transmission electron microscopy showing ultrastructure of MSCs and iHEPs (A). M = mitochondria; N = Nucleus; L = Lipids. Scale bars = 2 μm. Flow cytometry analysis of iHEPs, primary hepatocytes (HEPs) and MSCs stained with Mitotracker (B). MFI = Median fluorescence intensity.

**Additional file 3: Table S1.** Primer sequences used for plasmid cloning and gene expression analysis by RT-qPCR.

## Data Availability

The datasets used and/or analyzed during the current study are available from the corresponding author on reasonable request.

## Abbreviations

iHEPs: Hepatocyte-like cells
MSCs: Mesenchymal stromal cells
GFP: Green fluorescent protein
ITS: Insulin-transferrin-selenium
APAP: Acetaminophen
βNF: Naphthoflane
ICG: Indocianine green
LDL: Low-density protein
TTNPB: Arotinoid acid
AAT: Alpha 1 antitrypsin
AFP: Alpha-fetoprotein
CK18: Cytokeratin 18
CK19: Cytokeratin 19
E12.5: Mouse embryonic day 12.5
PAS: Periodic acid-Schiff
αSMA: Alpha-smooth muscle actin
EMT: Epithelial-mesenchymal transition
MM: Maturation media
iPSCs: Induced pluripotent stem cells
