# Supplementary figures and images for: Phenotype instability of hepatocyte-like cells produced by direct reprogramming of mesenchymal stromal cells
Source: Stem Cell Res Ther. 2020 Apr 10;11:154. doi: 10.1186/s13287-020-01665-z (PMC7323614; doi:10.1186/s13287-020-01665-z)

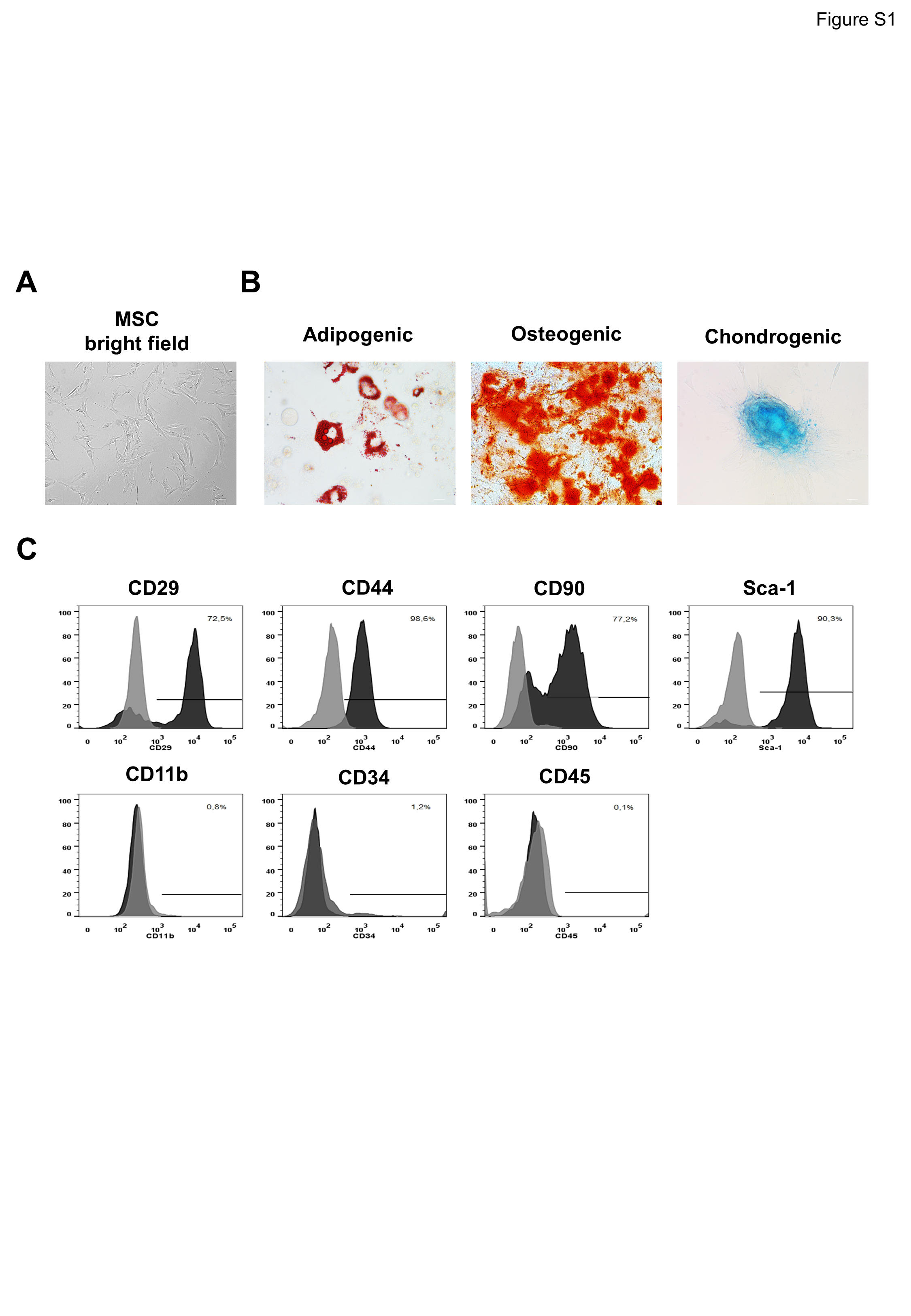

Supplement: Supplementary file 1 — Additional file 1 : Figure S1. Characterization of mesenchymal stromal cells. MSCs’ morphology seen by phase-contrast microscopy (A), and representative images of tri-lineage differentiation assays (B) showing positive staining for Oil Red (adipogenic), Alizarin Red (osteogenic) and Alcian Blue (chondrogenic). Flow cytometry analysis with a panel of MSCs and hematopoietic cell markers (C). [file 13287_2020_1665_MOESM1_ESM.bmp]

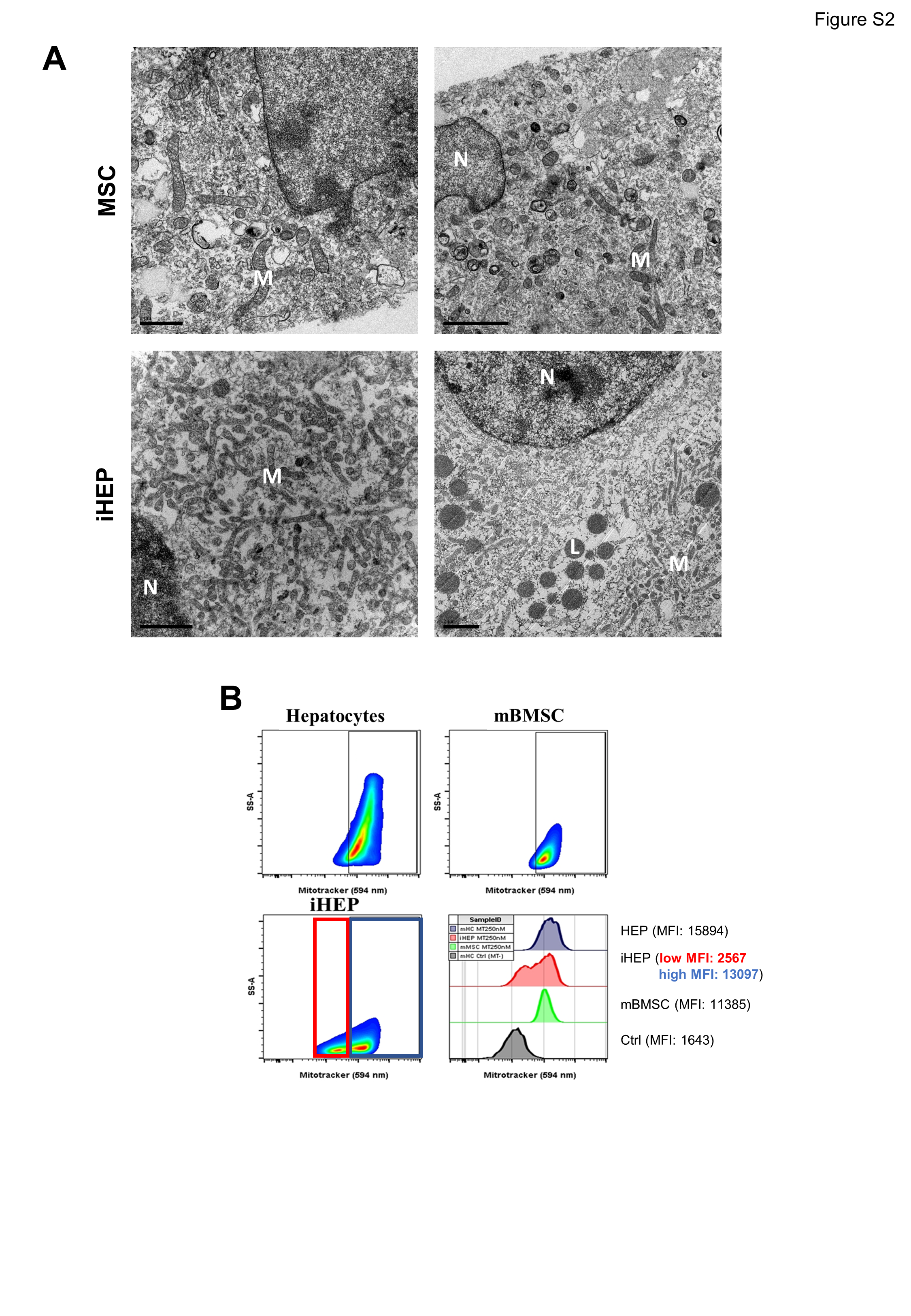

Supplement: Supplementary file 2 — Additional file 2 : Figure S2. Differences in mitochondrial content among iHEPs, MSCs and hepatocytes. Transmission electron microscopy showing ultrastructure of MSCs and iHEPs (A). M = mitochondria; N = Nucleus; L = Lipids. Scale bars = 2 μm. Flow cytometry analysis of iHEPs, primary hepatocytes (HEPs) and MSCs stained with Mitotracker (B). MFI = Median fluorescence intensity. [file 13287_2020_1665_MOESM2_ESM.bmp]
