# Supplementary material for: Phenotype instability of hepatocyte-like cells produced by direct reprogramming of mesenchymal stromal cells
Source: Stem Cell Res Ther. 2020 Apr 10;11:154. doi: 10.1186/s13287-020-01665-z (PMC7323614; doi:10.1186/s13287-020-01665-z)
Supplement: Supplementary file 3 — Additional file 3: Table S1. Primer sequences used for plasmid cloning and gene expression analysis by RT-qPCR. [file 13287_2020_1665_MOESM3_ESM.docx]

Table S1. Primer sequences used for plasmid cloning and gene expression analysis by RT-qPCR.

| **PRIMER** | **SEQUENCE** |
| --- | --- |
| **IRES-GFP_BamHI_F** | **ACTGGATCCCCATGCATCCAATTCCG** |
| **IRES-GFP_AscI_R** | **TTGGCGCGCCTTACTTGTACAGCTCGTC** |
| **mmHnf4a_XbaI_F** | **CATCTAGAATGCGACTCTCTAAAACCCTTG** |
| **mmHnf4a_BamHI_R** | **TTAGGATCCCTAGATGGCTTCTTGCT** |
| **mmFoxa2_BamHI_F** | **ATTAGGATCCATGCTGGGAGCCGTGAAG** |
| **mmFoxa2_BsrGI_R** | **ACGCCTGTACATTAGGATGAGTTCATAATAGG** |
| **mmAlbumin-F** | **TCGCTACACCCAGAAACCAC** |
| **mmAlbumin-R** | **CAGCAGACAACGCAG** |
| **mmAFP-F** | **ACTAGCGATGTGTTGGCTGC** |
| **mmAFP-R** | **ATGTGCTTTGCAACTCTCGG** |
| **mmEEF2 -F** | **TGAGATCAAGGACAGTGTGGTG** |
| **mmEEF2-R** | **TCATGAACATCAAACCGCACAC** |
| **mmTAT-F** | **TGTGAACAGCACTACCACTG** |
| **mmTAT-R** | **GTCCTTGCGTGGGACATGTC** |
| **mmAAT-F** | **CCTGCTAAACAGGCGCAGAA** |
| **mmAAT-R** | **TCGATGGTCAGCACAGCCTTA** |
| **mmCK18-F** | **GATCGTGGATGGCAGAGTGG** |
| **mmCK18-R** | **TTCCCTCCTTCTCTGCCTCAGT** |
| **mmCYP3A44-F** | **TGGACCCAGGAACTGCATTG** |
| **mmCYP3A44-R** | **GCATCCCGTGGCACAACTT** |
| **mmCYP3A11-F** | **TTCCAGCCTTGTAAGGAAACACA** |
| **mmCYP3A11-R** | **TGTACTGAATCTTTAACCAGGCATCA** |
| **mmCYP1A1 -F** | **CCTTCCGGCATTCATCCTTC** |
| **mmCYP1A1-R** | **TTTCAGGCCGGAACTCGTTT** |
| **mmBeta actin -F** | **TCCCTTGGATCTTTGCAGTT** |
| **mmBeta actin-R** | **CCCACAGCACTGTAGGGTTT** |
